# Pandemics and Multiple Crises in Latin America

**DOI:** 10.1111/lamp.12201

**Published:** 2020-11-30

**Authors:** Andrés Rivarola Puntigliano

## Introduction

The crisis caused by the Covid‐19 pandemic is certainly one of the worst in recent Latin American history. Forecasts for 2020 show a drop in the exports sector of approximately 23%, and a 9% reduction in gross domestic product (GDP). Unemployment is projected to rise from 8.1% in 2019 to 13.5% in 2020, and the poverty rate is expected to climb 7%, to 37.3% poverty is expected rise 4.5% (ECLAC, 2020a). The number of deaths due to Covid‐19 in the region has thus far reached more than 210,000 (Johns Hopkins University, 2020), and Latin America is clearly one of the most‐affected regions in the world.

As is known, this is not the first time the region has been affected by a pandemic. The initial European colonization was characterized by pandemics with devastating consequences for indigenous peoples. In more recent times we had the so‐called Spanish flu pandemic, from 1918 to the early 1920s, which is an interesting case to compare with the current situation. Figures on deaths at that time are not definitive, but there are estimates of approximately 40,000 in Chile, and of more than 12,000 in Argentina (López & Beltrán, 2013). An important similarity between the Spanish flu and Covid‐19 is how they uncovered the flaws of states with an inadequate health sector, as well as strong geographic and social asymmetries within states all over the world and between Latin American states (Carbonetti, 2010, p. 171). Then, as now, the fallout from the pandemic was a wake‐up call, making leading political actors and social forces more aware of the problems of social inequality and pushing demand for a more efficient overall state apparatus.

Although they are a century apart, another similarity between these two pandemics is the systemic juncture in which the crisis took place, which also showcases the weakness and vulnerability of Latin American states. By the early twentieth century, the region was heavily affected by World War I and the limitations of the external sector for economies heavily dependent on the export of commodities to Europe and the United States. Latin America was feeling the consequences not only of trade disruption due to a World War but of deep changes in the power structures of the international system. The pandemic crisis shined a spotlight on a broader crisis beyond the pandemic itself. It is what I refer to as “multiple crises,” the combination of economic, social, health, and geopolitical crises.

Yet, in spite of huge obstacles, Latin America emerged from the multiple crises of the early twentieth century with new ideas and initiatives, creating cultural and institutional tools to deal with future challenges. A similar outcome may result from the current pandemic crisis.

## The Multiple Crises of the Twentieth Century

The war that started in Europe in the early twentieth century (August 1914) had global repercussions such as the breakdown of the international balance of power, with disruptions to the global trade and payments system. It was a geopolitical crisis in which, as Victor Bulmer‐Thomas (2003, p. 152) points out, the old international economic order perished, but the new one was dangerously unstable. Regarding Latin America, it implied the end of British predominance and the shift from London to New York as a leading international financial center. It was a mixed blessing for Latin America, particularly for the smaller republics, since new lending was intertwined with U.S. foreign‐policy objectives that included increasing U.S. control of domestic affairs. The process was already underway before the war, in actions such as the Roosevelt Corollary to the Monroe Doctrine (Williams, 2012, p. 102).

On the economic front, the crisis presented serious problems for Latin America, manifested in changes to commodity markets. It meant shifts in demand curves, which reached a clearly negative low point during the global recession in 1920–1921, just as the health crisis of the Spanish flu swept through Latin America. In the case of the economic crisis, there was a long‐term problem of commodity oversupply and a slowing down of demand for primary‐product exports in the center (Bulmer‐Thomas, 2003, p. 157). The situation worsened with protectionism in the United States and parts of Europe. It is even said that the “America first” slogan originated at this time (Lynne, 2013).

The economic difficulties and the pandemic were accompanied by a social crisis that was already marked by the outbreak of the Mexican Revolution in 1910. Social groups demanding rights and a place at the commanding heights of state power challenged more and more the power of established elites. Urban workers, military forces, and new immigrant and peasant communities were leading forces. World War I gave way to the Interwar period, which was marked by the slowest growth rates of the century in the European countries (Bértola & Ocampo, 2012, p. 136), with negative repercussions on the entire global trade system. Revolution was in the air, fueled by the events in Russia in 1917.

In 1918, the same year that the Spanish flu pandemic broke out, there was a massive popular revolt in Córdoba, Argentina. It was probably one of the social ripple effects of the multiple crises, capturing the desires of new social groups opposed to old oligarchic structures. Students were at the front of this wave of protests, originally from Argentina but with a spillover to the rest of Latin America. In fact, the manifesto of the *Federación Universitaria de Cordoba* (at the center of the revolt) was a call to the “free men of ‘South America’” (FUC, 1918). What would result in the “Córdoba Reform” had tremendous regional effects on most Latin American universities. The agenda included labor unions and the establishment of new political parties with Latin American integration, such as the Popular Revolutionary American Alliance (APRA) in Peru. The social consequences of the pandemic bridged class differences, aligning future political leaders and intellectuals, such as the statesmen Rómulo Betancourt in Venezuela and Haya de la Torre in Peru, with the pueblo (Camacho Rodríguez, 2018, p. 31). Hence, the demands of the movement reached beyond the university, seeking solutions to the multiple crises—pushing for a democratization of society, economic reforms, a stronger role for the state, and increased Latin American unity.

Facing the negative juncture of the multiple crises, Latin American countries had to find a new way to position themselves in the international economy. The door was opened to forge new tools to surmount the crisis. As Bértola and Ocampo (2012) point out, of all the “new types of stakeholders that came onto the scene during this period, there was one which greatly expanded its sphere of influence”—the state (p. 137). In some countries the state had already taken on a leading role, promoting infant industries in the manufacturing sector, national banking systems, infrastructure, and natural‐resource‐based activities, and shaping labor and social‐development institutions.

Much of the early 1920s was a preamble to the ideas and political movements that would later identify with structuralism and developmentalism, leading to the creation of entities such as the United Nations Economic Commission for Latin America (ECLA; later renamed the United Nations Economic Commission for Latin America and the Caribbean—ECLAC) and the Inter‐American Development Bank (IDB). Both institutions advocated that without integration, there was no development. Latin America needed to unite through more‐efficient states to confront the crisis. Dealing with pandemics was part of a broader social concern for which development was needed. National and regional unity was imperative to confront this challenge, including dealing with the interests of great powers that prioritized their own interests rather than those of the region.

## The Multiple Crises of the Twenty‐first Century

After the so‐called commodity boom of the early twenty‐first century, Latin America suffered (with national and sub‐regional variations) a new economic downturn. Similar to the one in the early twentieth century, it was accompanied by a shift in the power structure of the global system, where we see the combination of an economic and global crisis in Latin America. Like in the early twentieth century, there is a pandemic crisis, Covid‐19, and a social crisis. Again, as before, besides its negative economic, social, and human consequences, the pandemic has made painfully evident the great vulnerabilities of Latin American states. More than create new problems, the pandemic has exacerbated existing ones, and there is no easy way out.

The current economic situation is bad and may worsen, but the downturn did not start with the Covid‐19 pandemic. Issues such as increasing indebtedness have been growing since the end of the so‐called commodity boom, the mid‐2010s, and the diminishing role of industrial exports (reprimarization) in favor of commodities started long before. The economic crisis is making painfully evident the traditional structural problem of the region, with dependence on commodity exports concentrated in a few markets. Indeed, exports go to more countries, but the bulk are still dependent on buyers from large markets.

Earlier advances toward industrialization had been eroded by the commodity boom, but fundamentally by decades of neoliberal reforms that dismantled the role of the state in the economy and in society. Regarding reprimarization, Brazil is a good example. After successful industrialization policies during the so‐called Brazilian miracle of the 1970s, primary goods again dominated Brazilian exports in the 2000s (Cardozo, 2018). In Mexico, the main issue was not reprimarization but a state losing its grip on its own territory. The incorporation of Mexico in the North American Free Trade Agreement (NAFTA) in the 1990s was positive in terms of industrializing Mexico’s exports, but at the cost of dismantling the state and national integration. As Morales (2008, p. 197) points out, Mexico was the laboratory of radical transformation, where NAFTA was used as the “hammer to chip away at state prerogatives” (McKinley, 2010, p. 96).

The economic crisis goes hand in hand with the social one. Although there were advances in poverty reduction in the early 2000s, the economic downturn is eroding these gains. Having started before the pandemic but certainly acerbated by it, these conditions will perhaps lead to a new “Lost Decade” in social terms. It is certainly not related only to reprimarization. In Mexico, “long‐run poverty barely declined since 1992 due to the high levels of inequality that persist in the country” (Iniguez Montiel & Kurosak, 2018, p. 23). As did the Spanish flu, Covid‐19 has had worse consequences for poorer sectors of the society (ECLAC, 2020a, p. 164, 168), leading to widening social asymmetries and increasing tensions.

The crises are linked and cannot be disconnected from the broader global geopolitical crisis that is creating problems and tensions for Latin American countries. In the early 1920s, there was tension due to the rivalry between the United States and Great Britain, and to a lesser degree, France. The 2020s are dominated by the U.S.–China rivalry, with intervention from less relevant powers such as Russia and the European Union. For Mexico it implies tensions between the power dominating its exports—the United States, with Mexican interests on the import side—, and China, which has gained more influence. In the case of Brazil, and most South American countries, China has become a pivotal—in some cases the most important—trade partner, for both imports and exports. China has even become a predominant source of financing for Latin American states.

The twenty‐first century interpretation of “America first” has placed Latin America in a new geopolitical situation. The new “America first” doctrine has not meant ignoring Latin America. On the contrary, “America” is applied to the continent, in the traditional geopolitical sense of U.S. foreign policy. After Barack Obama’s government (2009–2017) ended the Monroe Doctrine, the new administration of President Donald Trump rapidly reinstalled it. The argument was clear; as in the past, the Monroe Doctrine was invoked to reject what the United States defines as intervention by non‐continental powers. Warning against China and Russia, Secretary of State Rex Tillerson held that, “our region must be diligent to guard against far‐away powers that do not reflect the fundamental values shared in this region” (Brunnstrom et al., 2018). In spite of Cuba, the former rivalry with the Soviet Union did not entail a true threat to U.S. continental dominance, at least not as China does, which—along with Russia and other allies—has a much greater potential to challenge the United States (and the European Union) in traditional domains such as trade (including advanced technology) and the financial sector.

The shift from London to New York that took place during the first series of crises is mirrored in a current shift from New York to Beijing, although it is still too early to say if will be a complete shift, how long it may last, or the kind of conflicts it will entail. Still, a change is certainly occurring, and the geopolitical struggle is increasing the pressure on Latin American countries. An example in the trade sector is the imposition of steel tariffs on Brazil, and tariffs on Argentine agricultural commodities. Latin Americans have also been affected by U.S. directives to China and the European Union to buy its soy, impositions on Mexico regarding the control of migration flows from Central America, and pressure on several Latin American countries concerning diplomatic relations with Taiwan. In this case, “America” does not mean the continent but U.S. economic and political priorities.

As noted, the Covid‐19 pandemic has only made the situation more pronounced and highlighted new aspects, for example conflicts regarding the Amazonian region (Castro et al., 2020), or the power struggle concerning the acquisition of equipment to deal with the pandemic, such as respirators and facemasks. Powerful states set conditions and interfere in markets, to gain priority in acquiring equipment or to promote local companies. The power asymmetries are revealed in the rivalry over a Covid‐19 vaccine. As was the case with the crisis in the 1920s, the myth of free markets governing national and international relations showed to be a pipe dream. Companies and states join now to promote technology and control markets, where the role of the defense sector is of key importance in a geopolitical struggle led by great powers.

## Latin America, *Quo Vadis*?

What can the weaker states, such as those in Latin America, do? A good start would be to learn from the past, with the early 1920s as a good point of reference. We should pay attention to what the generation that was young at that time attempted to do later, in the 1950s and early 1960s—which was to apply “geopolitics of the weak.” It means regional integration where weaker states are grouped so that they can confront the larger ones. At least bargaining power should be improved, because power is the main currency of international trade. It was one of the lessons Raúl Prebisch learned as Argentina’s young representative to the 1933 International Monetary Conference in London, and later as General Secretary of ECLA (Dosman & Pollock, 1994, p. 21). Prebisch symbolizes a generation of brilliant experts and politicians who realized that there was an urgent need to abandon free‐trade‐oriented textbooks and assign a more active role to the state, to foster development and regional integration.

Fortunately, Latin Americans today do not need to walk in doctrinal *terra incognita*. There is a long tradition of action and thought based on developmentalism, and geopolitical insight and experience that can still be used. One of the main lessons is related to integration. The wise students from Córdoba understood that the way out of the crisis lay in cooperation within the region, so the appeal of their manifesto reached far beyond Argentina. Inspired by these early movements, the political forces promoting developmentalism held integration as a central goal.

It is still a main pledge of ECLAC. With the risk of turning the Covid‐19 health crisis into a food crisis, ECLAC’s Secretary General Alicia Bárcena is adamantly advocating for the urgency of regional cooperation that will outlast the pandemic. The call is, “for deepening regional integration through greater resilience in production networks, diversifying suppliers in terms of countries and companies, favoring locations that are closer to final consumption markets, and relocating strategic production‐related and technological processes” (ECLAC, 2020b). There are promising initiatives, such as collaboration between Mexico and Argentina regarding negotiations for a Covid‐19 vaccine, the Regional Contingency Plan elaborated by the Central American Integration System (SICA), and the Covid‐19 Observatory in Latin America and the Caribbean that resulted from the strategic alliance between the Community of Latin American and Caribbean States (CELAC) and ECLAC. Yet, much more is needed to cope with the dimension of the challenges.

This will not be the last pandemic, and multinational companies and the states in dominant positions are simply too big and too powerful for Latin American states to negotiate individually. Still, in the end, not everything is in the hands of states. As in the early 1920s, social movements and peoples need to take the initiative to promote national and regional agendas. Now, as then, the situation could be a benchmark to find solutions to national social crises in the midst of global ones, be they geopolitical, economic, or health challenges, or new ones such as climate change. Fragmentation was not the way to face structural challenges in the twentieth century, nor will it be in the twenty‐first century.
